# Fostering food safety culture in food service organizations: the synergistic role of spirituality and safety climate

**DOI:** 10.3389/fpubh.2026.1717466

**Published:** 2026-04-10

**Authors:** Xiujuan Chen

**Affiliations:** 1 School of Business, Jiangnan University, Wuxi, China; 2 Institute for Food Safety Risk Management, Jiangnan University, Wuxi, China

**Keywords:** food safety behavior, food safety climate, organizational culture, organizational psychology, organizational spirituality, spiritual leadership, workplace spirituality

## Abstract

Food safety remains a global public health challenge, yet traditional compliance-driven approaches often struggle to sustain genuine and consistent behavioral change. This perspective article examines the potential and limitations of integrating spirituality—across individual, workplace, and organizational levels—as a complementary force within food safety culture. Grounded in spiritual leadership theory and safety climate frameworks, we propose that spirituality may enhance food safety climate by fostering value alignment, ethical sensemaking, and shared responsibility. However, we acknowledge the conceptual and practical tensions inherent in applying spiritual constructs within often secular, highly regulated food safety systems—including measurement challenges, cultural and religious diversity, and the need for universally acceptable, evidence-based interventions. Notwithstanding these complexities, we argue that such integration warrants scholarly and practical exploration, particularly in an era marked by post-pandemic distrust, supply chain fragility, and regulatory fatigue. By synthesizing insights from organizational psychology, behavioral economics, and public health, this article advances a propositional thesis: that spirituality, if thoughtfully operationalized, could serve as a contributing factor in rebuilding trust, enhancing procedural integrity, and cultivating a more resilient and purpose-aware food safety culture. We conclude with a strategic agenda for future research and policy, calling for empirical validation, cross-disciplinary collaboration, and culturally adaptive approaches.

## 1 Introduction: Why spirituality, and why now?

Food safety remains a persistent global public health crisis (1), with recurring outbreaks, supply chain failures, and regulatory gaps undermining consumer trust and systemic resilience. As food safety culture is recognized as an effective strategy for enhancing food safety outcomes, the importance of fostering a positive food safety culture has gained increasing attention (2, 3). With the rise of the paradigm that “food safety equals behavior” (4), the food safety climate as a snapshot of food safety culture has become a key construct, which is defined as employees' (shared) perceptions of the current leadership, communication, commitment, resources, and risk awareness regarding food safety and hygiene in their work organizations (5). Research has shown that food safety culture, particularly food safety climate, exerts a critical influence on food handlers' behavior and safety outcomes (2, 6). A positive safety climate has been linked to improved food handling practices, reduced violations, and enhanced safety performance (2, 6, 7).

While research on food safety climate has advanced, prevailing theoretical and intervention models still pre-dominantly emphasize external regulatory mechanisms—such as compliance monitoring, audits, and punitive enforcement—to shape safety behaviors (2, 4). Although valuable, this compliance-centric approach may not sufficiently sustain employees' intrinsic motivation or adaptive behaviors for ensuring food safety, particularly in high-risk, relationship-intensive food service environments, where the limitations of purely external incentives become evident. Importantly, this is not to dismiss existing psychological perspectives; substantial work has examined constructs such as psychological safety, ethical leadership, moral identity, and psychological ownership in safety and organizational contexts (8, 9). However, these constructs—while capturing important facets of internal motivation—have rarely been systematically integrated into food safety climate frameworks in a way that addresses the deeper, holistic sense of meaning, purpose, and connectedness that can drive enduring behavioral internalization.

This gap points to the need for a more integrative perspective. We propose that spirituality–as a multi-dimensional construct encompassing personal meaning (individual spirituality), relational connections (workplace spirituality), and organizational purpose (organizational spirituality) (10)–can fundamentally enhance or reshape the food safety climate, promoting the internalization and practice of food safety culture. This is not a religious concept, but rather a broad pursuit of meaning, goals, connections, and ethical alignment in work by humans (11, 12). Its core lies in constructing an organizational culture that enables employees to view work beyond mere tasks, thereby enhancing wellbeing, trust, and performance (13, 14). Evidence from neuroscience, behavioral economics, and organizational psychology indicates that intrinsic motivation based on values is more enduring and adaptable than external reinforcement (15), yet this insight has not been systematically explored in food safety research, especially in high-risk food environments.

The selection of spirituality over related constructs such as moral identity, ethical leadership, or psychological ownership is deliberate and theoretically grounded. While each captures important facets of internal motivation, spirituality offers a more integrative framework that simultaneously operates at individual, relational, and organizational levels. Specifically, spirituality extends beyond existing models in three key ways: (1) unlike ethical leadership, which focuses pre-dominantly on leader behavior and role modeling, our framework considers spirituality as a multi-level phenomenon that operates through shared meaning, collective sensemaking, and institutional values; (2) whereas psychological safety captures team-level interpersonal trust, it does not account for deeper existential or value-based motivations—spirituality introduces the dimension of “meaningfulness” and “calling” that may enhance intrinsic adherence to safety protocols; and (3) while positive organizational behavior emphasizes psychological capacities such as hope and resilience, spirituality adds a normative and transcendent layer—connecting daily safety practices to broader purpose and moral responsibility. Thus, spirituality is not proposed as a substitute for these constructs but as a complementary and potentially catalytic dimension that addresses motivational and existential gaps not fully captured by existing models. In the context of food safety, where sustained vigilance requires not only personal integrity and leadership example but also collective purpose and interconnected responsibility, spirituality's multi-level architecture provides unique explanatory and influential potential that more circumscribed constructs cannot fully encompass.

This perspective article thus seeks to bridge the conceptual divide between “soft” organizational psychology and “hard” food safety science. We argue that the integration of spirituality into food safety culture is not only theoretically novel but also practically urgent, offering a pathway to rebuild trust, enhance procedural vigilance, and foster a culture of care in a food service industry increasingly characterized by fragmentation and distrust. We also argue that spirituality, operationalized in secular and inclusive terms, can serve as a catalytic, multilevel force that strengthens food safety climate by fostering deeper value alignment, shared ethical responsibility, and a culture of care. This integration responds to contemporary challenges—including post-pandemic distrust, workforce fragmentation, and regulatory fatigue—by offering a pathway to rebuild trust, enhance procedural vigilance, and cultivate a more resilient and purpose-oriented food safety culture.

## 2 A synergistic model: how spirituality transforms safety climate

This article mainly anchors its arguments in two complementary theoretical frameworks: safety climate theory and spiritual leadership theory. Safety climate theory (16, 17) provides a foundation for understanding the shared cognition (i.e., “climate”) that shapes employees' safe behaviors. It posits that employees interpret “what is truly important here” by observing organization's priorities, commitments, and procedures (i.e., “climate indicators”). However, traditional research on safety climate often focuses on extrinsic indicators such as policies and supervision, paying insufficient attention to the deeper, value-based intrinsic motivations (i.e., “why safety is important here”). This is precisely where spiritual leadership theory (18) offers a complementary perspective. The theory proposes that by fostering an organizational culture based on mission/vision, hope/belief, and altruistic love, leaders can evoke employees' intrinsic motivation and sense of meaning, thereby moving beyond mere compliance toward achieving exceptional organizational performance and ethical conduct.

**Figure 1:**
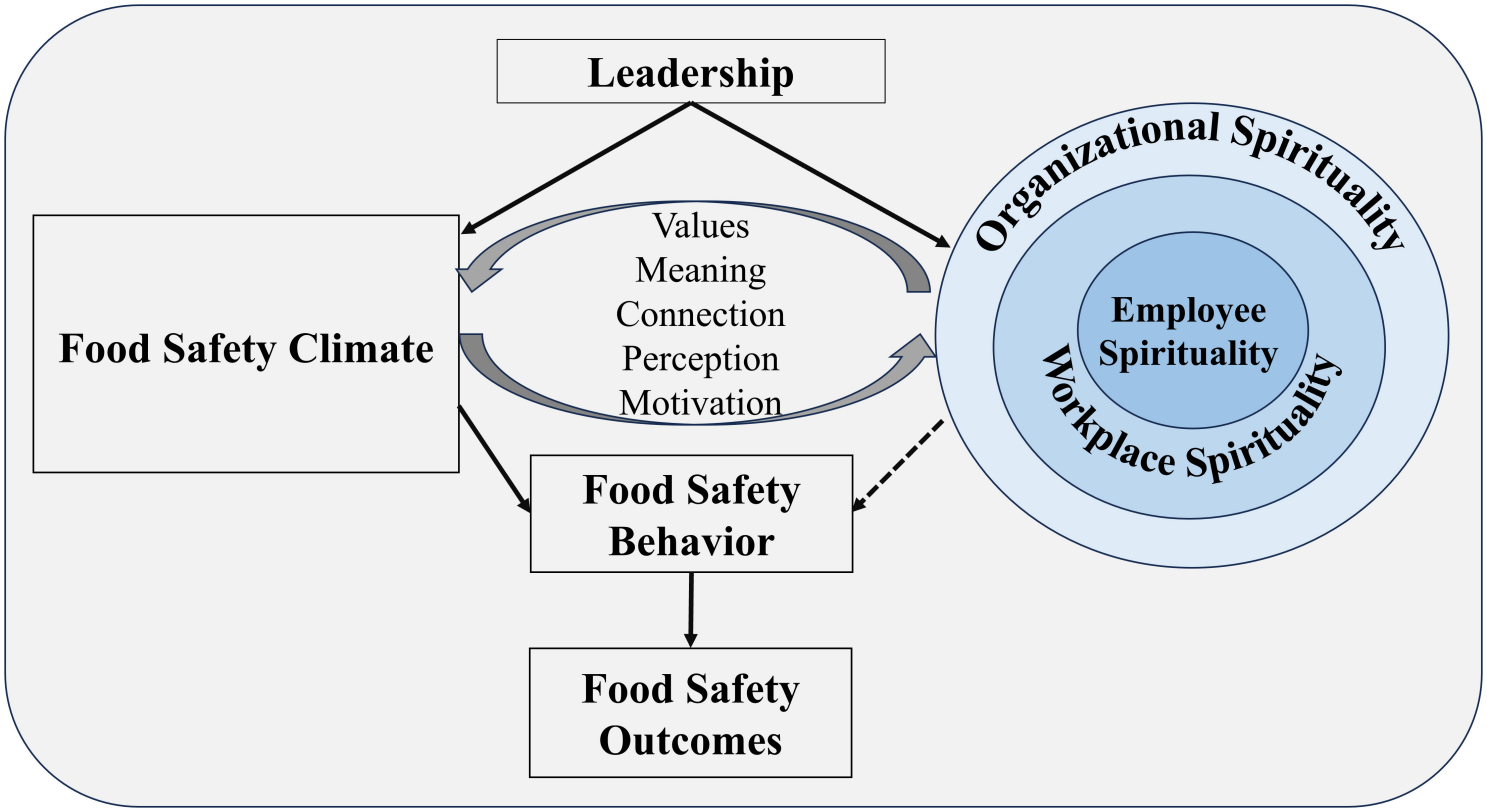
A synergistic multi-level model of spirituality and food safety climate. Arrows indicate reciprocal reinforcement mechanisms, reflecting the dynamic and co-constitutive relationship between spiritual constructs and climate perceptions, rather than unidirectional causality.

As shown in Figure 1, the interplay between spirituality and food safety climate can be conceptualized as a dynamic, multi-level system in which spiritual constructs at the individual, workplace, and organizational levels interact reciprocally with the perceptual and behavioral dimensions of food safety climate. This interaction is not linear but synergistic, creating a reinforcing cycle that shapes and sustains food safety culture. Below, we systematically outline this conceptualization using a multi-level framework. In this framework, spirituality at the individual, workplace, and organizational levels interacts with food safety climate through mechanisms of value internalization, motivation reinforcement, sensemaking, social cohesion, and cultural institutionalization (17, 19). This interaction may be dynamic, reciprocal, and potentially transformative, offering a holistic framework for cultivating food safety cultures that are not only compliant but also committed, not only procedural but also purposeful. To clearly explain under what conditions the different levels of spirituality might transform into safer food handling behaviors, this article distinguishes between the concepts of individual spirituality, workplace spirituality, and organizational spirituality as seen in Table 1.

**Table 1:** Distinction of multi-level spirituality.

Dimension	Individual spirituality	Workplace spirituality	Organizational spirituality
Essence	Personal inner state (14, 54)	Collective Experience in the Workplace (27, 55, 56)	System attributes of the organization (19)
Whether it depends on the existence of the organization	No (Exist independently) (14)	Yes (Depend on the job role and context) (27)	Yes (an organizational characteristic in itself) (19)
Origin	Personal inner pursuit (54)	Interaction between employees and their work/colleagues (55)	Conscious design, leadership and cultural shaping (18, 19)

### 2.1 Individual spirituality as the foundational lens of perception and motivation

In the current study, individual spirituality emphasizes an individual's inner experience and value orientation rather than specific religious doctrines or rituals (20, 21). Spirituality is also reflected in the inner driving forces of human beings, their pursuit of a sense of fulfillment and purpose in life (22). Within the framework of positive psychology, spirituality is often regarded as a personality advantage with the ability to shape behavior (23). The spirituality is a beneficial and productive resource for both personal wellbeing and the business goals of the organization (14, 24). It is operationalized through personal values alignment (e.g., viewing one's role as contributing to consumer wellbeing) and cognitive framing (e.g., perceiving food safety tasks as morally significant rather than merely procedural). In food safety, this translates into a personal ethic of care and responsibility. Employees with higher personal spirituality are more likely to become workers guided by values of service, mindfulness, and “doing the right thing”, and are more likely to adhere meticulously to hygiene protocols (25), report food contamination risks, and see their role as protecting consumer wellbeing, not merely following food safety rules.

From the perspective of cognitive evaluation theory, tasks aligned with personal values are more likely to stimulate autonomous motivation, and this motivation is crucial for improving food safety practices in the foodservice industry (15, 26). Food safety behaviors driven by individual spirituality may be most effective under the following organizational conditions: (1) When the organization's food safety climate clearly signals that “safety is a priority”, the internalization of employees' personal values is reinforced; (2) When the work design allows for a certain degree of autonomy rather than complete mechanical control, employees' moral identity can function effectively; (3) When employees perceive a visible connection between their individual contributions and consumer wellbeing (such as receiving consumer thanks), the motivational effect of meaning construction will be stronger. In a word, individual spirituality forms the foundational layer of both workplace spirituality and organizational spirituality. Functioning as a lens of perception and motivation, it enables employees to receive, interpret, and internalize the food safety climate.

### 2.2 Workplace spirituality as the relational and social mediator

Workplace spirituality refers to the shared experience of meaning, community, and transcendence among employees within a team or department (27, 28). Here, spirituality is treated as a unit-level, shared property that emerges through interaction, shared narratives, and relational norms rather than a simple aggregation of individual orientations (27). Workplace spirituality functions as a relational network that bridges individual spirituality and collective climate perceptions. Its role in shaping food safety climate can be explained through the lens of social learning theory (29) and team psychological safety. Essentially, workplace spirituality establishes a high-quality social interaction environment that fosters prosocial team norms and psychological safety. This, in turn, enables the genuine enactment of safety climate dimensions such as “peer commitment” and “open communication” (5, 30). Furthermore, workplace spirituality shapes social norms and offers social support, thereby translating individual motivations into collective behavioral patterns and ultimately enhancing food safety performance.

Workplace spirituality may play a greater role under the following organizational conditions: (1) when teams exhibit high stability and frequent interaction, fostering the development of deep relational trust; (2) when organizational structures are flattened or cross-hierarchical communication is encouraged, thereby reducing the communication barriers typically imposed by power distance; and (3) when formal mechanisms, such as regular team-reflection sessions and safety-partnership programs, systematically support team building and informal communication, thereby providing a “nurturing ground” for spiritual connections in the workplace.

### 2.3 Organizational spirituality as the cultural and strategic enabler

Organizational spirituality refers to an organizational culture guided by a mission statement, leadership, and business practices driven by social responsibility and higher-order values (21, 31). At this level, the referent is the organization; spirituality is evidenced by institutional features, including governance, resource allocation, and accountability systems that make purpose credible in practice (10). It emphasizes concern for the health and welfare of others, acknowledges employees' contributions to the organization, and promotes personal spiritual development and wellbeing (12, 31). Organizational spirituality is manifested in the organization's mission, values, leadership practices, and policies that prioritize higher-order goals—such as social responsibility and human flourishing—above mere profit (10, 12, 31). Organizational spirituality provides the cultural and strategic framework within which both safety climate and workplace spirituality are institutionalized. According to institutional theory, organizational spirituality embeds the pursuit of food safety values into the legitimizing normative structure of the organization, making it a core aspect of organizational identity. It serves as a resource for food practitioners in the workplace, ensuring that the pursuit of food safety is perceived as an integral part of the organizational purpose, rather than as a compliance burden. Conversely, a strong food safety climate fosters a shared sense of mission and ethical commitment (32), which in turn may help cultivate and stabilize organizational spirituality.

The potential mechanisms through which organizational spirituality contributes to enhancing food safety performance in food service organizations may include core processes such as value institutionalization and the shaping of leadership behaviors and decision-making frameworks. The impact of organizational spirituality is likely to be most pronounced under the following organizational conditions: (1) the leadership exhibits a high degree of value consistency and can demonstrate commitment to spiritual values through repeated communication and aligned decision-making; (2) the organization maintains transparent governance structures and accountability mechanisms, ensuring that resource commitments are fulfilled and safety performance is evaluated fairly; (3) the organization operates in an industry or market highly sensitive to its social reputation, making adherence to ethical standards, such as those pertaining to food safety, a strategic necessity for long-term organizational survival.

## 3 Integrating spirituality into fostering a positive food safety culture

The feasibility of integrating spirituality into the construction of food safety culture lies in transforming core spiritual concepts into specific leadership behaviors, structured employee interventions, and quantifiable assessment indicators, while making practical predictions and management of inherent obstacles in the food industry.

### 3.1 Critical considerations: ethical, measurement, and cultural challenges

While the integration of spirituality into food safety culture offers influential potential, it is imperative to engage critically with the conceptual and practical tensions that such integration entails. A growing body of skeptical scholarship has raised concerns about the managerial co-optation of spirituality, the risks of normative control, and the potential exclusion of non-spiritual or religiously diverse employees (33). These critiques caution that spirituality, if imposed top-down, may become a subtle yet powerful instrument of normative control—shaping employees' identities and values to align with organizational goals, thereby blurring the line between authentic engagement and manufactured consent (34). In highly regulated food service environments, where compliance pressures are already intense, the introduction of spiritual discourse could inadvertently intensify psychological expectations, leading to stress when employees fall short of value-laden ideals (35).

Measurement and construct validity present further challenges. Although instruments for workplace spirituality have proliferated, concerns persist regarding their psychometric robustness, discriminant validity, and susceptibility to social desirability bias (36). Spirituality, by its nature subjective and deeply personal, resists reduction to quantifiable indicators without loss of meaning. In food safety contexts—where metrics like pathogen counts and audit scores dominate performance evaluation—there is a risk that spirituality may be instrumentalized, valued only insofar as it predicts compliance outcomes, thereby undermining its intrinsic, humanistic essence (14).

Culturally, the transplantation of spirituality frameworks developed pre-dominantly in Western, Judeo-Christian contexts raises concerns about cultural imperialism and religious insensitivity (37). In multicultural food service teams, imposing a singular spiritual narrative may marginalize employees with secular worldviews or non-Western spiritual traditions, creating friction rather than cohesion. Even well-intentioned practices—such as values-based recognition or meaning-focused dialogues—must be carefully adapted to avoid privileging certain belief systems. As Lips-Wiersma and Mills (38) argue, workplace spirituality must remain “hospitable” to difference, fostering inclusion rather than enforcing conformity.

Acknowledging these critiques is not to dismiss the potential of spirituality, but to insist that its application be reflexive, context-sensitive, and ethically grounded. Therefore, any implementation strategy must prioritize voluntary participation, secular framing, cultural adaptation, and transparent dialogue about its purpose and limits.

### 3.2 Actionable strategies for practitioners and policymakers

Emphasizing the prominent role of leadership and the spiritual capital of leaders is the first key point for integrating spirituality into the development of a positive food safety culture. The organizational safety climate starts at the top and flows down from there (4, 16); organizational spirituality is similarly pioneered by leaders and built from the top down (Figure 1). The foundational role of leadership in shaping organizational culture and a food safety climate has been widely recognized (4, 39). Leaders should be responsible for establishing a good (excellent, if possible) overall food safety culture (4) and food safety climate (6), as important elements of food safety. A good food safety leader will inspire and encourage all personnel to work in a hygienic and food-safe manner (5). Leadership styles can influence commitment and create an ideal organizational climate and food safety culture (40). Sincere leaders serve others, make decisions based on their beliefs and values, and inspire the spirituality among employees and in the workplace, which is critical for promoting organizational spiritual development (10, 41). Moreover, integrating spirituality into leadership allows the entrepreneur's spiritual capital to align their actions, attitudes, and values, inspiring themselves and others through vision, hope, or faith and altruistic love (18), making employees feel that their work in the food service organization is meaningful.

Based on spiritual leadership theory, transforming spiritual capital into observable leadership behavior is the key to integration. To achieve spiritual integration, leaders need to go beyond traditional compliance regulations and adopt practical and empirically assessable spiritual leadership practices, including:

- Meaning-making and value narratives: Leaders can share the “Food Safety Mission Story” regularly (such as at monthly safety meetings), clearly linking daily operations (such as handwashing, temperature monitoring, and equipment calibration) with broader organizational goals (“Protecting the health of families in the community”, “ safeguarding brand reputation”) and universal values (care, responsibility). This practice, which aligns with the “stories to numbers” approach used successfully in aviation and healthcare safety culture transformation (42), imbues mundane tasks with deeper significance (18). In healthcare, similar narrative practices have been shown to improve staff engagement with safety protocols by connecting technical compliance to patient outcomes (43).
- Values-based recognition system: In addition to rewarding zero incidents, organizations can establish “Food Safety Values in Practice Awards” to publicly recognize employees who proactively report minor errors even when unsupervised, help colleagues correct unsafe behaviors, or propose innovative ideas for improving safety procedures. Award criteria shall be aligned with regulatory compliance indicators to avoid conflicts, shifting the focus from “result compliance” to “procedural integrity” and “proactive responsibility” (40). This approach mirrors the “just culture” principles adopted in high-reliability organizations, where reporting errors and near-misses is valued over concealing them (44).

Emphasizing the alignment of employee spiritual development with organizational spirituality and workplace spirituality is the second key point for integrating spirituality into the development of a positive food safety culture. Through the implementation of organizational spirituality, we aim to foster the individual spirituality of food processing employees and enhance their experience of workplace spirituality. Organizational spirituality is rooted in the values, practices, and discourse of workplace spirituality and personal spirituality, and influenced by environmental factors, knowledge management, and organizational culture (10). There must be consistency between the spirituality of individual members and the spirituality of the workplace and organization (45, 46); otherwise, conflicts and internal friction are likely to arise. The benefits of aligning members' personal spirituality with organizational spirituality include enhanced trust and psychological wellbeing among employees when they perceive that the organization practices higher-level values (31).

The synergy between personal spirituality and organizational/workplace spirituality is pivotal for the effectiveness of spiritual integration (45). To achieve effective synergy, we propose that design specific interventions connecting the individual and organizational levels:

- Value-oriented socialized and structured meaning dialogue mechanism: Add a module on “The Meaning of Our Work” in both new employee onboarding and annual refresher training. Moving beyond the rulebook, this module goes beyond regulatory explanations to include group discussions and dedicated sessions on the company's history, value-driven stories, and real-world cases of ethical food safety practices. It emphasizes “why we do things this way” and invites colleagues of different lengths of service to share their most proud moments related to food safety in their careers. This approach helps internalize safety norms and construct a shared narrative of meaning (10). However, meaning-oriented dialogue may alienate employees with secular worldviews or different spiritual traditions. If mandatory or linked to performance evaluation, it risks becoming normative control rather than authentic engagemen (36, 38).
  Design spiritually friendly physical and social environment: this can be operationalized as follows: (a) Setting up “Safety Values Walls” in areas such as break rooms, changing rooms, or corridor walls, displaying photos of employees and their families alongside slogans such as “I follow procedures to keep my family safe”, and excerpts from customer thank-you letters or positive feedback mentioning food safety, thereby strengthening emotional connections—a practice analogous to healthcare's use of patient stories to reinforce the purpose of safety protocols (43); (b) Establishing a “peer mentoring” system, with senior employees serving as “safety partners” for new recruits. They not only impart skills but also share their understanding of food safety culture and provide social support. This mirrors Crew Resource Management (CRM) in aviation, which flattened hierarchies and empowered all team members to speak up on safety matters (47); (c) Allowing teams to hold small reflection and celebration ceremonies after completing major safety improvement projects or achieving a long-term record of no food safety incidents, to honor collective achievements and strengthen the sense of belonging to “our common success”.
- Integrate spiritual dimensions into food safety communication and training: In food safety risk communication, alongside presenting data (e.g., incidence rates of specific pathogens), incorporate emotional and value-based appeals (e.g., “A single oversight may cause significant suffering to multiple consumers, which contradicts our core value of caring for others”). In training, case studies can help participants explore how to make value-aligned safety decisions under pressure, using secular language centered on meaning, responsibility, care, and integrity to avoid religious connotations. However, to address potential tensions with secular regulatory frameworks, these practices must remain voluntary, clearly separated from formal performance appraisal, and reviewed for legal and regulatory neutrality. This approach also helps mitigate the unintended consequence of normative bias—the assumption that spiritual employees are better employees, which risks marginalizing those with different perspectives (36).

### 3.3 Potential barriers to implementing spirituality-based approaches

It must be acknowledged that implementing spirituality-based approaches in highly regulated and risk-averse industries such as food safety, to foster a positive food safety culture aimed at enhancing food safety outcomes, faces some significant barriers:

Firstly, traditional regulatory compliance pressures and short-termism: In highly regulated industries such as food safety, management attention is often focused on avoiding immediate pressures such as penalties, potentially viewing the cultivation of spirituality as a “soft” or “long-term” investment and thus allocating insufficient resources (6). Introducing an intrinsic motivation framework based on values may be regarded as a secondary or redundant core compliance requirement. To address this, leaders must define spirituality as a cultural enhancer of technological compliance (not a substitute), embed spiritual interventions into the safety training to reduce additional burdens, and develop short-term indicators (e.g., improved training engagement) to demonstrate immediate value.

Secondly, challenges in measurement and standardization: while food safety metrics are readily quantifiable (e.g., pathogen counts or recall incidents), the benefits of spiritual capital—such as enhanced trust, resilience, or intrinsic vigilance—tend to be qualitative and lagging indicators. This poses a challenge for ROI- focused management in cost-sensitive operational environments. This barrier can be partially mitigated by adopting mixed-methods frameworks that combine validated scales (5, 28) with qualitative interviews and objective safety outcomes. However, the deeper issue concerns whether inherently subjective experiences such as spirituality can—or should—be standardized within management systems designed for uniformity and predictability.

Thirdly, conceptual misunderstanding and skepticism—a fundamental theoretical challenge: Employees or managers may misinterpret “workplace spirituality” as introducing religious content or dismiss it as a managerial “gimmick.” Successful implementation depends on clear, secularized communication that is closely linked to the core business (food safety), emphasizing its relevance to “meaning in work, values, and mutual respect” (27) rather than to religious belief systems. However, spirituality, by its nature, resists codification: what provides meaning and purpose to one employee may hold little resonance for another. This raises a critical question: can personal meaning-making coexist with non-negotiable, evidence-based safety protocols?

The resolution of this tension does not lie in forcing a standardized “spirituality” onto employees, but in creating an organizational environment where personal meaning is derived from the very act of upholding universal standards. The “meaning” for an employee is not found in interpreting the protocol differently, but in understanding that their precise, unwavering adherence to it is an act of profound care—for the consumer, for their colleagues, and for the integrity of their craft. The standardization of the outcome (safe food) becomes the canvas upon which individuals paint their personal sense of purpose. For instance, a line worker might find meaning in being a “guardian of public health,” a quality controller in being a “protector of the vulnerable.” Thus, the coexistence is possible not by making protocols negotiable, but by framing strict compliance as a vehicle for expressing personal and shared values. The challenge for leadership is to build a culture that consistently connects the “what” (the rigid protocol) to the “why” (the meaningful impact), allowing individual purpose to flourish within the boundaries of collective discipline. This transforms the protocol from a mere bureaucratic imposition into a source of professional and spiritual pride.

## 4 Discussion and future research suggestions

Spirituality, once regarded as a peripheral topic, is now recognized as fundamental to understanding employee motivation, ethical behavior, and organizational resilience (14, 27). Both spirituality and food safety climate address shared themes—such as employee psychological perceptions and values—which are closely linked to improved organizational performance outcomes (31, 48, 49). While existing models of food safety culture acknowledge the importance of food safety climate (5, 50), they lack a framework to integrate the profound dimension of spirituality. The rationale for introducing spirituality in this perspective article is twofold: (1) to address the potential need for deeper, value-driven motivators in food safety culture that go beyond cognitive and behavioral compliance; and (2) to respond to the growing academic and practical recognition of holistic, human-centered spiritual approaches in organizational management.

By integrating insights from organizational psychology, safety science, and spirituality research, this paper proposes a novel framework for advancing food safety culture—one that acknowledges human spirituality as a significant resource in the pursuit of safer food systems. While this perspective highlights the potential value of incorporating spirituality into the food safety culture framework, we acknowledge that the proposed links between spiritual constructs, food safety climate, and performance are theoretically derived and still require rigorous empirical validation. This perspective is therefore offered as an invitation to scholarly debate and empirical inquiry, rather than as a prescriptive model or established pathway.

Future research employing cross-sectional surveys, longitudinal designs, or quasi-experimental interventions is needed to infer their associations, establish causality, and examine the evolution of these constructs over time. To advance from conceptual advocacy to scientific validation, we propose the following theoretically grounded hypotheses derived directly from our multi-level framework:

H1: individual spirituality positively moderates the relationship between food safety climate perceptions and food safety behavior, such that the climate-behavior link is stronger for employees with higher levels of personal spirituality.

H2: workplace spirituality mediates the relationship between organizational spirituality and team-level food safety climate, such that organizations with higher institutional spirituality foster stronger team-level spiritual climates, which in turn enhance shared perceptions of safety priorities.

H3: the positive effect of spiritual leadership on food safety compliance is sequentially mediated by (a) employees' sense of meaning at work and (b) their internalization of safety-related values.

Testing these hypotheses requires research designs capable of capturing both the phenomenological depth of spirituality and its link to objective safety outcomes. We recommend mixed-methods approaches including: (1) sequential explanatory designs combining quantitative surveys of spiritual constructs and food safety climate with in-depth phenomenological interviews of purposefully sampled employees; (2) ethnographic case studies in high-risk food service environments (e.g., hospital kitchens, cruise ship catering) to observe how spiritual narratives and social interactions shape safety behaviors; (3) daily diary methods capturing fluctuations in meaning-making and their proximal relationship to safety vigilance; and (4) integration of qualitative and survey data with objective indicators such as hygiene audit scores, pathogen testing results, and incident reports.

Furthermore, future research is needed to explore how to foster a positive food safety climate and a more stable, sustainable workplace/organizational spirituality across different cultural contexts, thereby motivating spontaneous safety behaviors among organizational members and ultimately enhancing the performance and competitiveness of food enterprises. Recognizing the limitations of a simplistic East-West dichotomy, future inquiry should adopt a more nuanced approach to cultural applicability, particularly given the prevalence of multicultural workplaces in the food service industry. Rather than treating cultures as monolithic national or regional blocks, researchers should consider the dynamic interplay of individual and collective value orientations within the same organizational setting. For example:

- In contexts where individualistic cognitive frames are salient—whether among employees in North America, Western Europe, or within diverse teams globally—spirituality may emphasize personal meaning, self-actualization, and autonomous responsibility. Food safety behaviors could be framed as expressions of individual professionalism and intrinsic values. Promotion strategies in such contexts should focus on discourse such as “personal empowerment” and “conscientious fulfillment.”
- Conversely, where collectivist value orientations are more prominent—such as in East Asia, Latin America, or among employees from these cultural backgrounds working in multicultural teams—spirituality tends to align with team harmony, family responsibility, and organizational loyalty. Food safety can be shaped as a moral obligation of “safeguarding the collective” or “living up to the trust placed in us.” Emphasizing concepts like “the wellbeing of the community” and “collective honor” is likely to be more effective.

Future studies should also investigate how these cultural frames interact in multicultural work environments, where employees from diverse backgrounds may negotiate shared spiritual or value-based commitments to food safety. Understanding such intercultural dynamics will be critical for developing flexible, inclusive, and context-sensitive interventions that resonate across the cultural spectrum present in modern food enterprises.

Moreover, although the measurement of spirituality is becoming increasingly feasible (51), given that spirituality is essentially subjective and abstract, the measurement of spiritual culture is still challenging due to social approval bias, cultural universality issues, and the multi-level complexity of spirituality (27, 36, 52). Future studies should prioritize careful construct specification, tests of discriminant validity, and measurement invariance across contexts, rather than relying on single-source self-reports alone (53). At the same time, incorporating spiritual aspects into the discussion framework of food safety organizational culture may unintentionally marginalize employees without spiritual beliefs or confuse personal beliefs with professional compliance. That is, organizational spirituality discourse may pose the risk of normative bias. Ensuring that management practices are inclusive, ethical, and foster a sense of personal responsibility among employees may help contribute to the construction of a food safety culture in food service organizations.

Integrating spirituality into the construction of food safety culture requires moving from vague conceptual advocacy to concrete, actionable, and evaluable leadership practices and organizational interventions. Despite challenges stemming from industry-specific constraints, measurement difficulties, and cultural inertia, the careful design of evidence-based interventions—such as structured meaning-focused dialogues, values-based recognition systems, and spiritually informed leadership training—and the empirical validation of their effectiveness can provide a solid practical pathway and theoretical foundation for this emerging field. This approach holds the potential to foster more intrinsic, enduring, and proactive food safety behaviors.

## Data Availability

The original contributions presented in the study are included in the article/supplementary material, further inquiries can be directed to the corresponding author.
